# Impact of early use of long acting paliperidone (1 and 3 monthly) in a first-episode psychosis sample

**DOI:** 10.1192/j.eurpsy.2021.2144

**Published:** 2021-08-13

**Authors:** P. Gil Lopez, J.M. Rodriguez, L. Garcia Fernandez, E. Fernandez Martin, N. Fernandez Gayoso, V. Sanchez Estevez

**Affiliations:** Early Intervention Service For First Psychotic Episodes “lehenak”, Mental Health Network of Bizkaia (RSMB). Basque Health System. Osakidetza, Bilbao, Spain

**Keywords:** outcome, Relapse, first-episode psychosis, long-acting paliperidone

## Abstract

**Introduction:**

Relapse prevention is a key objetive for patients with a First Episode Psychosis (FEP) and the low adherence to antipsychotic (AP) treatment is the main reason for relapse after a FEP.

**Objectives:**

There are no clear recommendations about the early use of long-acting injectables (LAIs) in FEP. We review the impact on hospitalization rates of the early use (earlier than 1 year after the inclusion in our Early Intervention Service “Lehenak”) of LAI paliperidone in a FEP sample.

**Methods:**

We evaluated in a naturalistic study a sample (N=384) of patients with a FEP. We carried out a mirror-design study to compare the numer of hospitalizations before and after the introduction of LAI paliperidone (1 and 3 monthly) in early users (<1 year) vs late users (>1 year).

**Results:**

A total of 384 FEP patients with LAI paliperidone were assessed.

**Table:** 

	Early Paliperidone LAI (n=201)	Late Paliperidone LAI (n=173)	Within groups comparisons t (p)
Hospitalizations pre-LAI mirror period (media, standard deviation)	1.76 (1.97)	2.22 (2.60)	1,87 (0.06)
Days in hospital pre-LAI mirror period	21.42 (28.28)	28.02 (38.27)	1.87 (0.06)
Hospitalizations post-LAI mirror period	0.68 (1.61)	0.80 (1.74)	0.73 (0.46)
Days in hospital post-LAI mirror period	15.17 (40.58)	18.78 (45.24)	0.81 (0.42)

**Conclusions:**

There was no difference between the early and late introduction of LAI Paliperidone in the number of hospitalizations after treatament. There was a trend to present more previous hospitalizations and days in hospital in late users. This could support an earlier use of paliperidone LAI to prevent an excess of hospitalizations due to late introduction.

**Disclosure:**

The presenting author has received honouraria for lectures or advisory boards from Janssen, Otsuka, Lundbeck and Angelini in the last five years.

